# 2-Isopropyl-5-methyl­cyclo­hexyl cyclo­hex­yl(phen­yl)phosphinate

**DOI:** 10.1107/S1600536811010592

**Published:** 2011-03-31

**Authors:** Hao Xu, Li-Juan Liu, Fan-Jie Meng, Chang-Qiu Zhao

**Affiliations:** a College of Chemistry and Chemical Engineering, Liaocheng University, Shandong 252059, People’s Republic of China

## Abstract

In the title mol­ecule, C_22_H_35_O_2_P, the two cyclo­hexyl rings exhibit chair conformations. In the crystal, mol­ecules related by translation along the *b* axis are linked by the weak inter­molecular C—H⋯O hydrogen bonds.

## Related literature

For the crystal structure of a related P-chiral compound, see: Fu & Zhao (2010 ▶).
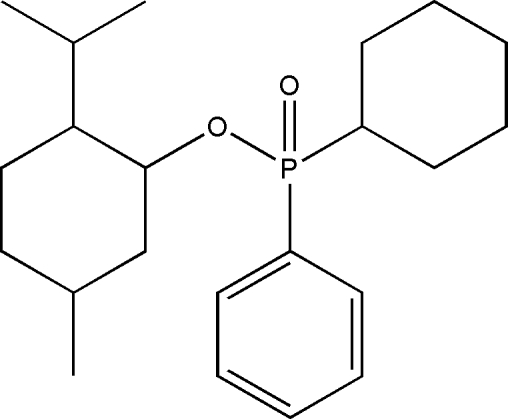


## Experimental

### 

#### Crystal data

C_22_H_35_O_2_P
*M* *_r_* = 362.47
Monoclinic, 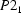

*a* = 11.4892 (13) Å
*b* = 5.8872 (6) Å
*c* = 16.3531 (17) Å
β = 94.696 (1)°
*V* = 1102.4 (2) Å^3^
*Z* = 2
Mo *K*α radiation
μ = 0.14 mm^−1^
*T* = 298 K
0.45 × 0.11 × 0.08 mm

#### Data collection

Bruker SMART-1000 CCD area-detector diffractometer
Absorption correction: multi-scan (*SADABS*; Sheldrick, 1996 ▶) *T*_min_ = 0.941, *T*_max_ = 0.989
5833 measured reflections
3646 independent reflections
2125 reflections with *I* > 2σ(*I*)
*R*_int_ = 0.051

#### Refinement

*R*[*F* ^2^ > 2σ(*F* ^2^)] = 0.053
*wR*(*F* ^2^) = 0.119
*S* = 0.92
3646 reflections
229 parameters
1 restraint
H-atom parameters constrained
Δρ_max_ = 0.15 e Å^−3^
Δρ_min_ = −0.16 e Å^−3^
Absolute structure: Flack (1983 ▶), 1493 Friedel pairs
Flack parameter: 0.19 (14)

### 

Data collection: *SMART* (Bruker, 2007 ▶); cell refinement: *SAINT* (Bruker, 2007 ▶); data reduction: *SAINT*; program(s) used to solve structure: *SHELXS97* (Sheldrick, 2008 ▶); program(s) used to refine structure: *SHELXL97* (Sheldrick, 2008 ▶); molecular graphics: *SHELXTL* (Sheldrick, 2008 ▶); software used to prepare material for publication: *SHELXTL*.

## Supplementary Material

Crystal structure: contains datablocks I, global. DOI: 10.1107/S1600536811010592/cv5058sup1.cif

Structure factors: contains datablocks I. DOI: 10.1107/S1600536811010592/cv5058Isup2.hkl

Additional supplementary materials:  crystallographic information; 3D view; checkCIF report

## Figures and Tables

**Table 1:** Hydrogen-bond geometry (Å, °)

*D*—H⋯*A*	*D*—H	H⋯*A*	*D*⋯*A*	*D*—H⋯*A*
C21—H21*A*⋯O2^i^	0.97	2.54	3.376 (5)	145
C17—H17⋯O2^i^	0.98	2.47	3.346 (5)	149

Symmetry code: (i) 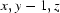.

**Fig. 1.**
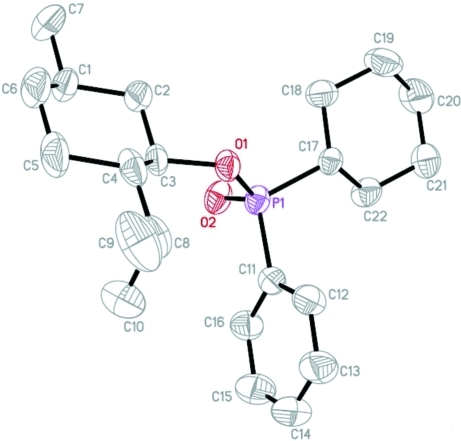
The molecular structure of (I) showing the atomic numbering and 50% probability displacement ellipsoids. H atoms have been omitted for clarity.

## supplementary crystallographic information

### Comment

In continuation of our study of phenylphosphinates (Fu & Zhao, 2010), we present here the title compound (I) prepared from substitution of *O*-menthyl phenylphosphoryl chloride with cyclohexyl magnesium chloride.

In (I) (Fig. 1), the configuration of phosphorus atom was determined as *R*. The compound is comprised of fully extended substituents: cyclohexyl, menthyoxy and phenyl, which form an irregular tetrahedron. The geometric parameters of (I) are usual. The bond angle of C17—P—C11 is 108.65 (18)°, O1—P—C11 is 105.37 (15)°, O1—P—C17 is 102.47 (15)°, O2—P—O1 is 113.94 (16)°, O2—P—C17 is 115.03 (18)° and O2—P—C11 is 110.65 (18) °.

In the crystal structure, the molecules related by translation along *b* axis are linked by the weak intermolecular C—H···O hydrogen bonds (Table 1) into chains.

### Experimental

*O*-Menthyl phenylphosphoryl chloride (0.3 mmol) was added to a stirred ether solution of cyclohexyl magnesium chloride (0.6 mmol) under nitrogen and the mixture was stirred for 24 h at room temperature. After washing with water, the resulting solution was purified by preparative TLC on silica gel to afford optically pure product. The crystal suitable for X-ray diffraction was obtained from recrystallization with ethyl ether/hexane.

### Refinement

All H atoms were fixed geometrically and treated as riding, with C—H = 0.93 - 0.98 Å and with *U*_iso_(H) = 1.2-1.5 *U*_eq_(C).

### Crystal data

**Table:**

C_22_H_35_O_2_P	*F*(000) = 396
*M**_r_* = 362.47	*D*_x_ = 1.092 Mg m^−^^3^
Monoclinic, *P*2_1_	Mo *K*α radiation, λ = 0.71073 Å
Hall symbol: P 2yb	Cell parameters from 1233 reflections
*a* = 11.4892 (13) Å	θ = 2.5–26.0°
*b* = 5.8872 (6) Å	µ = 0.14 mm^−^^1^
*c* = 16.3531 (17) Å	*T* = 298 K
β = 94.696 (1)°	Block, colourless
*V* = 1102.4 (2) Å^3^	0.45 × 0.11 × 0.08 mm
*Z* = 2	

### Data collection

**Table:**

Bruker SMART-1000 CCD area-detector diffractometer	3646 independent reflections
Radiation source: fine-focus sealed tube	2125 reflections with *I* > 2σ(*I*)
graphite	*R*_int_ = 0.051
φ and ω scans	θ_max_ = 25.0°, θ_min_ = 2.5°
Absorption correction: multi-scan (*SADABS*; Sheldrick, 1996)	*h* = −13→10
*T*_min_ = 0.941, *T*_max_ = 0.989	*k* = −6→6
5833 measured reflections	*l* = −19→19

### Refinement

**Table:**

Refinement on *F*^2^	Secondary atom site location: difference Fourier map
Least-squares matrix: full	Hydrogen site location: inferred from neighbouring sites
*R*[*F*^2^ > 2σ(*F*^2^)] = 0.053	H-atom parameters constrained
*wR*(*F*^2^) = 0.119	*w* = 1/[σ^2^(*F*_o_^2^) + (0.0454*P*)^2^] where *P* = (*F*_o_^2^ + 2*F*_c_^2^)/3
*S* = 0.92	(Δ/σ)_max_ < 0.001
3646 reflections	Δρ_max_ = 0.15 e Å^−^^3^
229 parameters	Δρ_min_ = −0.16 e Å^−^^3^
1 restraint	Absolute structure: Flack (1983), 1493 Friedel pairs
Primary atom site location: structure-invariant direct methods	Flack parameter: 0.19 (14)

### Special details

**Table:**

Geometry. All e.s.d.'s (except the e.s.d. in the dihedral angle between two l.s. planes) are estimated using the full covariance matrix. The cell e.s.d.'s are taken into account individually in the estimation of e.s.d.'s in distances, angles and torsion angles; correlations between e.s.d.'s in cell parameters are only used when they are defined by crystal symmetry. An approximate (isotropic) treatment of cell e.s.d.'s is used for estimating e.s.d.'s involving l.s. planes.
Refinement. Refinement of *F*^2^ against ALL reflections. The weighted *R*-factor *wR* and goodness of fit *S* are based on *F*^2^, conventional *R*-factors *R* are based on *F*, with *F* set to zero for negative *F*^2^. The threshold expression of *F*^2^ > σ(*F*^2^) is used only for calculating *R*-factors(gt) *etc*. and is not relevant to the choice of reflections for refinement. *R*-factors based on *F*^2^ are statistically about twice as large as those based on *F*, and *R*- factors based on ALL data will be even larger.

### Fractional atomic coordinates and isotropic or equivalent isotropic displacement parameters (Å^2^)

**Table:**

	*x*	*y*	*z*	*U*_iso_*/*U*_eq_	
O1	0.23767 (17)	0.3099 (5)	0.23127 (13)	0.0631 (7)	
O2	0.0655 (2)	0.5689 (4)	0.26004 (16)	0.0743 (8)	
P1	0.11424 (7)	0.33939 (18)	0.26861 (5)	0.0574 (3)	
C1	0.3668 (4)	0.8147 (10)	0.1266 (3)	0.1001 (15)	
H1	0.3503	0.9307	0.1671	0.120*	
C2	0.2885 (3)	0.6094 (8)	0.1401 (2)	0.0784 (13)	
H2A	0.2073	0.6561	0.1330	0.094*	
H2B	0.3008	0.4947	0.0991	0.094*	
C3	0.3131 (3)	0.5077 (7)	0.2245 (2)	0.0656 (11)	
H3	0.2941	0.6204	0.2654	0.079*	
C4	0.4397 (3)	0.4344 (8)	0.2429 (3)	0.0788 (13)	
H4	0.4556	0.3199	0.2017	0.095*	
C5	0.5175 (4)	0.6387 (9)	0.2281 (3)	0.1134 (18)	
H5A	0.5059	0.7535	0.2692	0.136*	
H5B	0.5986	0.5913	0.2351	0.136*	
C6	0.4939 (4)	0.7439 (10)	0.1434 (3)	0.118 (2)	
H6A	0.5436	0.8759	0.1392	0.141*	
H6B	0.5136	0.6351	0.1022	0.141*	
C7	0.3404 (5)	0.9182 (10)	0.0410 (3)	0.141 (2)	
H7A	0.3626	0.8129	0.0003	0.211*	
H7B	0.3837	1.0566	0.0371	0.211*	
H7C	0.2583	0.9498	0.0322	0.211*	
C8	0.4657 (3)	0.3225 (10)	0.3271 (3)	0.0899 (13)	
H8	0.4093	0.1986	0.3305	0.108*	
C9	0.5883 (4)	0.2143 (10)	0.3369 (4)	0.138 (2)	
H9A	0.6015	0.1294	0.2884	0.207*	
H9B	0.5936	0.1145	0.3835	0.207*	
H9C	0.6462	0.3316	0.3450	0.207*	
C10	0.4503 (4)	0.4809 (11)	0.4004 (3)	0.1130 (18)	
H10A	0.5076	0.5998	0.4014	0.170*	
H10B	0.4603	0.3955	0.4505	0.170*	
H10C	0.3735	0.5462	0.3950	0.170*	
C11	0.1394 (3)	0.2629 (7)	0.3751 (2)	0.0579 (11)	
C12	0.1898 (3)	0.0589 (8)	0.4006 (3)	0.0777 (12)	
H12	0.2096	−0.0463	0.3617	0.093*	
C13	0.2111 (4)	0.0089 (9)	0.4834 (3)	0.0985 (16)	
H13	0.2465	−0.1278	0.4995	0.118*	
C14	0.1804 (5)	0.1593 (12)	0.5412 (3)	0.1094 (18)	
H14	0.1933	0.1251	0.5967	0.131*	
C15	0.1302 (4)	0.3623 (12)	0.5164 (3)	0.1083 (17)	
H15	0.1102	0.4666	0.5556	0.130*	
C16	0.1088 (3)	0.4143 (8)	0.4334 (2)	0.0821 (14)	
H16	0.0738	0.5516	0.4176	0.099*	
C17	0.0280 (3)	0.1208 (6)	0.2167 (2)	0.0535 (9)	
H17	0.0700	−0.0232	0.2254	0.064*	
C18	0.0112 (4)	0.1628 (9)	0.1254 (2)	0.0875 (14)	
H18A	0.0867	0.1661	0.1029	0.105*	
H18B	−0.0256	0.3095	0.1153	0.105*	
C19	−0.0646 (4)	−0.0220 (11)	0.0820 (2)	0.1093 (17)	
H19A	−0.0772	0.0142	0.0241	0.131*	
H19B	−0.0238	−0.1662	0.0871	0.131*	
C20	−0.1814 (4)	−0.0440 (10)	0.1178 (3)	0.1077 (17)	
H20A	−0.2260	0.0944	0.1077	0.129*	
H20B	−0.2251	−0.1684	0.0913	0.129*	
C21	−0.1641 (3)	−0.0873 (8)	0.2087 (3)	0.0850 (15)	
H21A	−0.1264	−0.2334	0.2183	0.102*	
H21B	−0.2396	−0.0931	0.2312	0.102*	
C22	−0.0900 (3)	0.0966 (8)	0.2526 (2)	0.0755 (13)	
H22A	−0.1313	0.2402	0.2478	0.091*	
H22B	−0.0776	0.0593	0.3104	0.091*	

### Atomic displacement parameters (Å^2^)

**Table:**

	*U*^11^	*U*^22^	*U*^33^	*U*^12^	*U*^13^	*U*^23^
O1	0.0564 (13)	0.0584 (18)	0.0767 (15)	−0.0131 (15)	0.0192 (11)	−0.0118 (15)
O2	0.0739 (17)	0.055 (2)	0.096 (2)	0.0069 (16)	0.0223 (14)	0.0092 (15)
P1	0.0537 (5)	0.0531 (6)	0.0667 (6)	−0.0016 (6)	0.0136 (4)	0.0013 (6)
C1	0.118 (4)	0.091 (4)	0.098 (3)	−0.049 (4)	0.050 (3)	−0.018 (3)
C2	0.081 (3)	0.080 (3)	0.078 (3)	−0.022 (3)	0.025 (2)	−0.011 (2)
C3	0.064 (3)	0.057 (3)	0.078 (3)	−0.024 (2)	0.021 (2)	−0.018 (2)
C4	0.053 (3)	0.083 (3)	0.102 (3)	−0.020 (2)	0.016 (2)	−0.037 (3)
C5	0.079 (3)	0.119 (5)	0.146 (5)	−0.050 (3)	0.031 (3)	−0.034 (4)
C6	0.106 (4)	0.126 (5)	0.130 (5)	−0.055 (3)	0.060 (3)	−0.032 (4)
C7	0.193 (6)	0.128 (6)	0.109 (4)	−0.060 (5)	0.062 (4)	0.013 (4)
C8	0.063 (2)	0.088 (3)	0.116 (4)	−0.007 (3)	−0.013 (2)	−0.017 (4)
C9	0.083 (3)	0.115 (5)	0.208 (6)	0.013 (3)	−0.039 (4)	−0.046 (4)
C10	0.110 (4)	0.134 (5)	0.093 (3)	0.004 (3)	−0.004 (3)	−0.026 (4)
C11	0.051 (2)	0.063 (3)	0.061 (2)	−0.0008 (19)	0.0085 (18)	−0.005 (2)
C12	0.088 (3)	0.076 (3)	0.068 (3)	0.005 (3)	−0.003 (2)	−0.007 (2)
C13	0.112 (4)	0.099 (4)	0.080 (3)	0.007 (3)	−0.024 (3)	0.011 (3)
C14	0.129 (4)	0.132 (6)	0.065 (3)	−0.011 (4)	−0.003 (3)	−0.001 (4)
C15	0.131 (4)	0.125 (6)	0.072 (3)	0.005 (4)	0.028 (3)	−0.023 (4)
C16	0.090 (3)	0.088 (4)	0.071 (3)	0.001 (3)	0.021 (2)	−0.014 (3)
C17	0.047 (2)	0.053 (2)	0.062 (2)	−0.0024 (18)	0.0107 (17)	0.0018 (19)
C18	0.085 (3)	0.116 (4)	0.061 (3)	−0.025 (3)	0.007 (2)	0.000 (3)
C19	0.104 (4)	0.156 (5)	0.067 (3)	−0.027 (3)	−0.001 (3)	−0.023 (3)
C20	0.074 (3)	0.140 (5)	0.104 (4)	−0.027 (3)	−0.019 (3)	−0.012 (3)
C21	0.053 (2)	0.096 (4)	0.106 (3)	−0.017 (2)	0.003 (2)	−0.003 (3)
C22	0.052 (2)	0.101 (4)	0.074 (3)	−0.011 (3)	0.0116 (19)	0.007 (3)

### Geometric parameters (Å, °)

**Table:**

O1—C3	1.461 (4)	C10—H10A	0.9600
O1—P1	1.599 (2)	C10—H10B	0.9600
O2—P1	1.465 (3)	C10—H10C	0.9600
P1—C17	1.794 (4)	C11—C16	1.372 (5)
P1—C11	1.799 (4)	C11—C12	1.382 (5)
C1—C6	1.523 (6)	C12—C13	1.388 (5)
C1—C2	1.533 (6)	C12—H12	0.9300
C1—C7	1.534 (6)	C13—C14	1.362 (7)
C1—H1	0.9800	C13—H13	0.9300
C2—C3	1.510 (5)	C14—C15	1.374 (7)
C2—H2A	0.9700	C14—H14	0.9300
C2—H2B	0.9700	C15—C16	1.393 (6)
C3—C4	1.523 (5)	C15—H15	0.9300
C3—H3	0.9800	C16—H16	0.9300
C4—C5	1.529 (6)	C17—C18	1.511 (4)
C4—C8	1.535 (6)	C17—C22	1.528 (4)
C4—H4	0.9800	C17—H17	0.9800
C5—C6	1.521 (6)	C18—C19	1.530 (6)
C5—H5A	0.9700	C18—H18A	0.9700
C5—H5B	0.9700	C18—H18B	0.9700
C6—H6A	0.9700	C19—C20	1.513 (5)
C6—H6B	0.9700	C19—H19A	0.9700
C7—H7A	0.9600	C19—H19B	0.9700
C7—H7B	0.9600	C20—C21	1.506 (5)
C7—H7C	0.9600	C20—H20A	0.9700
C8—C10	1.540 (6)	C20—H20B	0.9700
C8—C9	1.542 (6)	C21—C22	1.520 (5)
C8—H8	0.9800	C21—H21A	0.9700
C9—H9A	0.9600	C21—H21B	0.9700
C9—H9B	0.9600	C22—H22A	0.9700
C9—H9C	0.9600	C22—H22B	0.9700
			
C3—O1—P1	119.5 (2)	C8—C10—H10A	109.5
O2—P1—O1	114.04 (15)	C8—C10—H10B	109.5
O2—P1—C17	115.11 (17)	H10A—C10—H10B	109.5
O1—P1—C17	102.36 (14)	C8—C10—H10C	109.5
O2—P1—C11	110.69 (17)	H10A—C10—H10C	109.5
O1—P1—C11	105.39 (14)	H10B—C10—H10C	109.5
C17—P1—C11	108.49 (17)	C16—C11—C12	118.6 (4)
C6—C1—C2	108.9 (4)	C16—C11—P1	118.7 (3)
C6—C1—C7	112.8 (4)	C12—C11—P1	122.7 (3)
C2—C1—C7	111.7 (4)	C11—C12—C13	121.0 (4)
C6—C1—H1	107.7	C11—C12—H12	119.5
C2—C1—H1	107.7	C13—C12—H12	119.5
C7—C1—H1	107.7	C14—C13—C12	120.2 (5)
C3—C2—C1	112.1 (3)	C14—C13—H13	119.9
C3—C2—H2A	109.2	C12—C13—H13	119.9
C1—C2—H2A	109.2	C13—C14—C15	119.1 (5)
C3—C2—H2B	109.2	C13—C14—H14	120.4
C1—C2—H2B	109.2	C15—C14—H14	120.4
H2A—C2—H2B	107.9	C14—C15—C16	121.0 (5)
O1—C3—C2	108.5 (3)	C14—C15—H15	119.5
O1—C3—C4	108.8 (3)	C16—C15—H15	119.5
C2—C3—C4	113.4 (3)	C11—C16—C15	119.9 (4)
O1—C3—H3	108.7	C11—C16—H16	120.0
C2—C3—H3	108.7	C15—C16—H16	120.0
C4—C3—H3	108.7	C18—C17—C22	110.4 (3)
C3—C4—C5	107.9 (4)	C18—C17—P1	111.8 (3)
C3—C4—C8	114.3 (3)	C22—C17—P1	111.0 (2)
C5—C4—C8	114.0 (4)	C18—C17—H17	107.8
C3—C4—H4	106.7	C22—C17—H17	107.8
C5—C4—H4	106.7	P1—C17—H17	107.8
C8—C4—H4	106.7	C17—C18—C19	111.3 (4)
C6—C5—C4	113.7 (4)	C17—C18—H18A	109.4
C6—C5—H5A	108.8	C19—C18—H18A	109.4
C4—C5—H5A	108.8	C17—C18—H18B	109.4
C6—C5—H5B	108.8	C19—C18—H18B	109.4
C4—C5—H5B	108.8	H18A—C18—H18B	108.0
H5A—C5—H5B	107.7	C20—C19—C18	111.8 (4)
C1—C6—C5	111.9 (4)	C20—C19—H19A	109.3
C1—C6—H6A	109.2	C18—C19—H19A	109.3
C5—C6—H6A	109.2	C20—C19—H19B	109.3
C1—C6—H6B	109.2	C18—C19—H19B	109.3
C5—C6—H6B	109.2	H19A—C19—H19B	107.9
H6A—C6—H6B	107.9	C21—C20—C19	110.3 (3)
C1—C7—H7A	109.5	C21—C20—H20A	109.6
C1—C7—H7B	109.5	C19—C20—H20A	109.6
H7A—C7—H7B	109.5	C21—C20—H20B	109.6
C1—C7—H7C	109.5	C19—C20—H20B	109.6
H7A—C7—H7C	109.5	H20A—C20—H20B	108.1
H7B—C7—H7C	109.5	C20—C21—C22	111.7 (4)
C4—C8—C10	114.4 (4)	C20—C21—H21A	109.3
C4—C8—C9	112.3 (4)	C22—C21—H21A	109.3
C10—C8—C9	109.3 (4)	C20—C21—H21B	109.3
C4—C8—H8	106.8	C22—C21—H21B	109.3
C10—C8—H8	106.8	H21A—C21—H21B	108.0
C9—C8—H8	106.8	C17—C22—C21	111.5 (3)
C8—C9—H9A	109.5	C17—C22—H22A	109.3
C8—C9—H9B	109.5	C21—C22—H22A	109.3
H9A—C9—H9B	109.5	C17—C22—H22B	109.3
C8—C9—H9C	109.5	C21—C22—H22B	109.3
H9A—C9—H9C	109.5	H22A—C22—H22B	108.0
H9B—C9—H9C	109.5		
			
C3—O1—P1—O2	25.3 (3)	O2—P1—C11—C12	−177.1 (3)
C3—O1—P1—C17	150.3 (3)	O1—P1—C11—C12	−53.3 (3)
C3—O1—P1—C11	−96.4 (3)	C17—P1—C11—C12	55.7 (3)
C6—C1—C2—C3	55.2 (5)	C16—C11—C12—C13	−1.2 (6)
C7—C1—C2—C3	−179.5 (4)	P1—C11—C12—C13	178.0 (3)
P1—O1—C3—C2	−93.0 (3)	C11—C12—C13—C14	1.3 (7)
P1—O1—C3—C4	143.1 (3)	C12—C13—C14—C15	−1.2 (8)
C1—C2—C3—O1	−178.3 (3)	C13—C14—C15—C16	1.0 (8)
C1—C2—C3—C4	−57.3 (5)	C12—C11—C16—C15	0.9 (6)
O1—C3—C4—C5	175.1 (3)	P1—C11—C16—C15	−178.2 (3)
C2—C3—C4—C5	54.3 (5)	C14—C15—C16—C11	−0.9 (7)
O1—C3—C4—C8	−57.0 (4)	O2—P1—C17—C18	60.0 (3)
C2—C3—C4—C8	−177.9 (4)	O1—P1—C17—C18	−64.3 (3)
C3—C4—C5—C6	−53.9 (5)	C11—P1—C17—C18	−175.4 (3)
C8—C4—C5—C6	178.1 (4)	O2—P1—C17—C22	−63.8 (3)
C2—C1—C6—C5	−54.5 (5)	O1—P1—C17—C22	171.9 (3)
C7—C1—C6—C5	−179.2 (4)	C11—P1—C17—C22	60.8 (3)
C4—C5—C6—C1	56.4 (6)	C22—C17—C18—C19	−54.7 (5)
C3—C4—C8—C10	−64.6 (5)	P1—C17—C18—C19	−178.8 (3)
C5—C4—C8—C10	60.1 (5)	C17—C18—C19—C20	55.9 (5)
C3—C4—C8—C9	170.0 (4)	C18—C19—C20—C21	−55.8 (6)
C5—C4—C8—C9	−65.3 (5)	C19—C20—C21—C22	56.0 (5)
O2—P1—C11—C16	2.0 (3)	C18—C17—C22—C21	55.1 (4)
O1—P1—C11—C16	125.8 (3)	P1—C17—C22—C21	179.7 (3)
C17—P1—C11—C16	−125.2 (3)	C20—C21—C22—C17	−56.2 (5)

### Hydrogen-bond geometry (Å, °)

**Table:**

*D*—H···*A*	*D*—H	H···*A*	*D*···*A*	*D*—H···*A*
C21—H21A···O2^i^	0.97	2.54	3.376 (5)	145
C17—H17···O2^i^	0.98	2.47	3.346 (5)	149

Symmetry codes: (i) *x*, *y*−1, *z*.
